# Daily COVID-19 symptom assessment over 28 days – findings from a daily direct-to-patient registry of COVID-19 positive patients

**DOI:** 10.1186/s41687-023-00668-7

**Published:** 2023-12-07

**Authors:** Emma Brinkley, Kendall Knuth, Tom Kwon, Christina Mack, Heidi Leister-Tebbe, Weihand Bao, Matthew W Reynolds, Nancy Dreyer

**Affiliations:** 1 IQVIA, 2400 Ellis Road, Durham, NC 27703 USA; 2 Pfizer Inc, 500 Arcola Road, Collegeville, PA 19426 USA; 3 Pfizer Inc, 66 Hudson Boulevard West, New York, NY 10001 USA; 4 328 Country Club Road, Newton, MA 02459 USA

**Keywords:** Real World evidence, COVID-19, Patient reported outcomes, Registry

## Abstract

In January 2021, 999 COVID-19 positive adults in the US enrolled in an online, direct-to-patient registry to describe daily symptom severity and progression over 28 days. The most commonly reported and persistent symptoms were fatigue, headache, decreased sense of taste, decreased sense of smell, and cough. Fast resolving symptoms included gastrointestinal symptoms (nausea, vomiting, diarrhea) and those related to fever and chills. While more than half (56%) of patients reported overall symptom improvement during the 28-day study period, 60% of patients were still reporting at least 1 COVID-19 symptom at the end of 28 days. Risk factors for experiencing symptoms for longer duration included at least one of the following: older age (> 60 years), higher BMI, lung disease, and receiving medication for hypertension. The study demonstrates the value of patient-reported data to provide important and timely insights to COVID-19 disease and symptom progression and the potential of using real-world data to inform clinical trial design and endpoints.

**Supplementary Information:**

The online version contains supplementary material available at 10.1186/s41687-023-00668-7.

## Background

As of December 2022, there have been over 645 million confirmed cases worldwide, including 6.6 million deaths [1]. COVID-19 presents itself with a variety of symptoms, including flu-like, respiratory, gastrointestinal, and neurological manifestations. Evidence suggests that duration of symptoms varies from days to months, with some patients experiencing long-term sequalae after the acute infection phase.

The objective of this study was to use an online, U.S., direct-to-patient registry to describe patient-reported daily COVID-19 symptom severity and progression for 28 days among symptomatic, non-hospitalized adults to help inform study design and clinical trial endpoints for an oral antiviral candidate. Early in the pandemic, there were no established or approved treatment options for COVID-19. This study was undertaken to support the aggressive timeless needed for treatment development by evaluating defined endpoints to inform subsequent sample size calculations needed to evaluate the efficacy of potential COVID-19 treatments in clinical trial.

## Materials and methods

We leveraged data from the Daily Symptom Sub-study of the IQVIA COVID-19 Active Research Experience (CARE, see www.helpstopcovid19.com). CARE was an online, direct-to-patient registry studying adults with COVID-19 infection, COVID-19 exposure, or vaccinated against COVID-19 [2–5]. Enrollment and data collection was closed in February 2023. Patients are recruited via social media targeting adult U.S. residents and self-reported demographics, COVID-19 testing, COVID-19 vaccinations, symptoms, medical encounters, and medical history including diagnoses. In this manuscript, we refer to these participants as patients, even if they may not have sought care.

Once enrolled in the CARE Study, patients were invited during January 2021 to participate in this sub-study if they had reported testing positive for SARS-SoV-2 infection (molecular (PCR test) or antigen test) and if they were symptomatic with at least one COVID-19 related symptom with onset of symptoms within 7 days before enrollment. Patients were asked to record symptom presence and severity at approximately the same time daily for 28 days after the baseline survey at enrollment. Patients received compensation of up to $60 for completing daily surveys, aggregated at the end of the 4-week survey.

Fifteen pre-defined COVID-19 related symptoms were measured including fever, chills, cough, fatigue, shortness of breath, headache, aches and pains, decreased sense of taste, decreased sense of smell, nasal congestion, runny nose, sore throat, nausea, vomiting, and diarrhea. Symptoms were selected based on COVID-19 symptoms reported by the US Food and Drug Administration in September 2020. Severity was assessed by patients using a 4-point scale (very mild, mild, moderate, severe). Symptom progression was measured as time to improvement and time to complete resolution, by individual symptoms and by overall symptoms. Last value carried forward was used for severity on days where participants failed to complete the severity in days 7, 14, 21, and 28.

### Data analysis

The numbers (n) and percentages (%) of patients reporting symptoms on each day during the 28 days of study were reported. Time to symptom improvement was defined as time from symptom onset to the first reduction in symptom severity without worsening during the study period (e.g., severe symptom at baseline reported as mild during follow-up). Symptom resolution was defined as time from symptom onset to complete resolution of symptoms without recurrence during the study period. Each symptom was assessed independently for reduction and resolution.

Proportional hazard Cox models were used to evaluate relationship between time to improvement for presence of all symptoms and potential covariates/factors. All COVID-19 symptoms, medical history, COVID-19 treatments, and type of COVID-19 test were included in the univariate analysis. The multivariable HRs were obtained by fitting a multivariable Cox regression model that included the subset of variables with univariate HRs p-values < 0.2. Results were presented as hazard ratios (HRs) with the corresponding 95% confidence intervals (CIs).

## Results

### Study patients and characteristics

A total of 999 CARE patients, who completed at least one survey, and reported a positive COVID-19 test result within 14 days prior to enrollment, were enrolled January of 2021 and included in the analysis. The median [interquartile range (IQR)] time between symptom onset and enrollment into the study was 5 [3; 6] days, and the median [IQR] time between positive test and enrollment into the study was 3 [2; 4] days.

The majority of patients were female (86%) and White (82%); 15% were Hispanic/Latino. The median age was 38 years (IQR: 30, 48). Almost half (49%) of patients were obese and 10% reported they currently were smokers. The most commonly reported co-morbidities for which they indicated they were using medications were hypertension (15%) and lung disease (6%). Mental health disorders were prevalent, with 31% reporting depression and 42% anxiety; 23% reported insomnia. Around 9% reported having received a COVID-19 vaccine at enrollment. Of the 999 patients, 737 (73.8%) completed the full 28 days of follow-up. The average follow-up contribution was 23 days.

### Daily presence of symptoms

The median [IQR] number of symptoms reported was 7 [5, 9] at enrollment and 1 [0, 3] on day 28. The median [IQR] number of moderate or severe symptoms at enrollment was 3 [2, 5] and 0 [0, 1] on day 28.

**Fig. 1:**
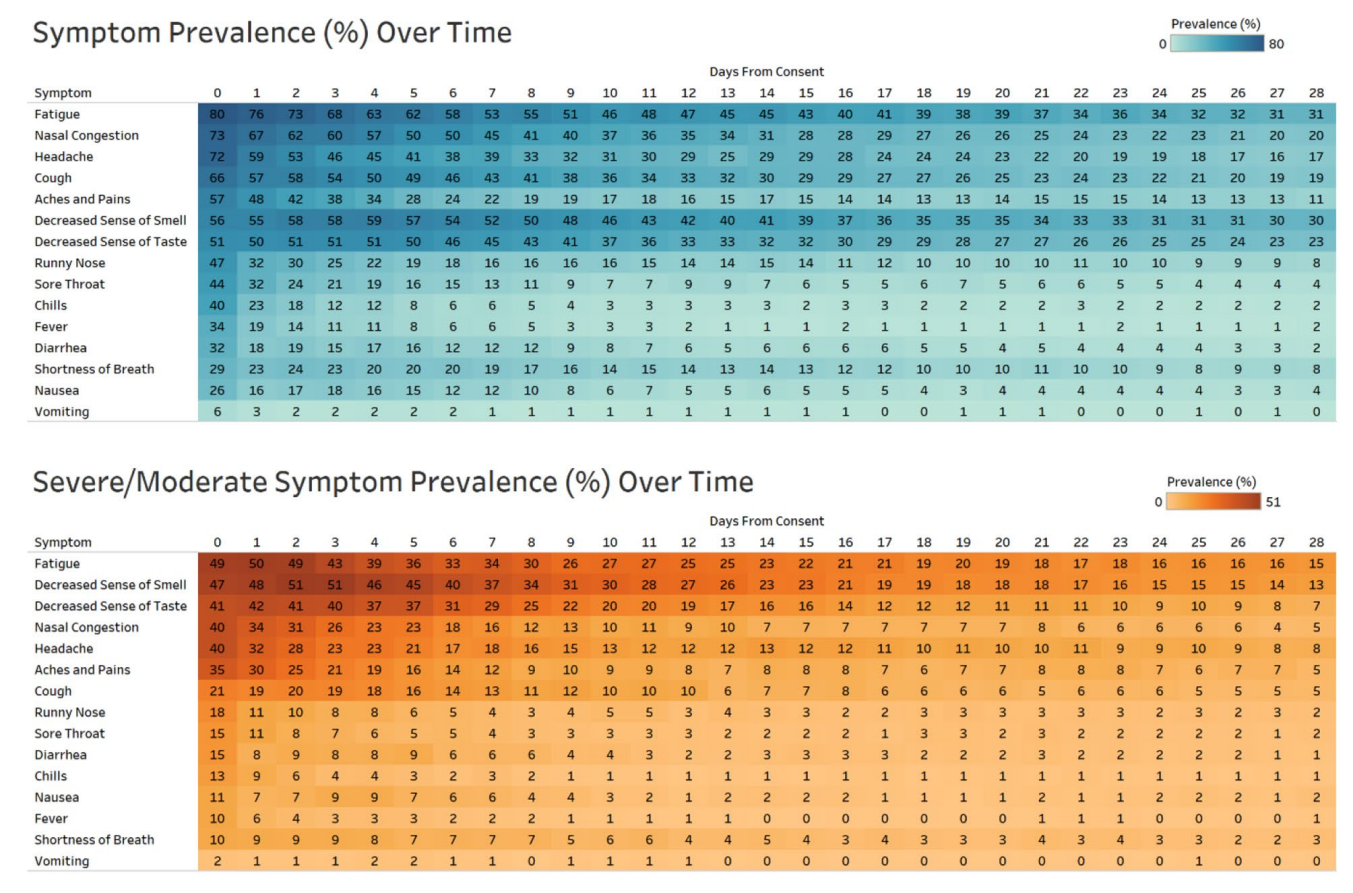
Self-reported COVID-19 Symptom Prevalence during 28 Day Follow-up

The proportions of patients reporting presence of each symptom daily during the study period are presented in Fig. 1, for any symptom and moderate-to-severe symptoms.

Fatigue (80%), nasal congestion (73%), headache (72%), cough (66%), aches and pains (58%), and decreased sense of taste (51%) and smell (56%) were highly prevalent at enrollment and were most persistent with approximately 10–30% still reporting these specific symptoms on day 28 of the study (Fig. 1). Chills (40%), sore throat (44%), fever (34%), diarrhea (32%), and nausea (26%) were also frequently reported at enrollment, but prevalence of these symptoms resolved more quickly, with fewer than 10% reporting these symptoms after 9 days.

### Time to symptom improvement and resolution

Just over half (56%) of patients reported improvement, without subsequent worsening, across all symptoms with 30% reporting complete resolution of all symptoms during the study period. During the study period, 43% reported returning to their normal health, defined by patient’s judgment, with a median [IQR] time to event of 28 [15; -] days.

### Time to symptom improvement and resolution: individual symptoms

Median time to improvement and resolution are presented in Table 1. For all symptoms, the median time to improvement was within 2 weeks of enrollment, with the median time to resolution within about 3 weeks of enrollment.

**Table 1:** Median time to COVID-19 symptom improvement and resolution

Symptom	Median [IQR] time to improvement in days	Median [IQR] time to resolution in days
Fatigue	14 [5;27]	22 [9;32]
Headache	11 [4;23]	18 [7;27]
Decreased sense of smell	10 [5;24]	19 [7;]
Cough	9 [4;18]	15 [6;28]
Decreased sense of taste	8 [4;17]	14 [6;]
Nasal congestion	8 [4;18]	15 [7;28]
Shortness of breath	7 [3;17]	11 [4;23]
Aches and pains	6 [3;19]	9 [4;25]
Runny nose	5 [2;13]	7 [3;20]
Sore throat	5 [2;12]	6 [3;15]
Nausea	4 [2;10]	5 [2;14]
Diarrhea	4 [2;10]	6 [2;15]
Fever	3 [2;6]	3 [2;7]
Chills	3 [2;6]	4 [2;9]
Vomiting	2 [2;4]	2 [2;6]

In the two weeks prior to enrollment, 33% of patients reported a medical encounter related to COVID-19. During the 28-day study period, 24% reported COVID-19 related medical encounter, including 15% reporting a telehealth visit, 5% reporting office visits, and < 1% reporting an emergency room visit.

### Multivariate cox proportional hazard modeling on overall presence of symptoms

Age, gender, BMI, smoking, lung disease, receiving medication for autoimmune disease, diabetes, and/or hypertension, taking an antibiotic, corticosteroid, or NSAID, and type of COVID-19 test were included in the multivariate Cox proportional hazard model. Older age (≥ 60 years vs. < 60 years; HR = 0.73, 95% CI: 0.50–1.07), higher body mass index (BMI) (overweight: HR = 0.76, 95% CI: 0.60–0.95; obese: HR = 0.76, 95% CI: 0.61–0.95; severe obesity: HR = 0.60, 95% CI: 0.45–0.81), lung disease (HR = 0.61, 95% CI: 0.43–0.85), and receiving medication for hypertension (HR = 0.70, 95% CI: 0.53–0.92) are related with lower likelihood of symptom improvement. Males had higher likelihood of symptom improvement compared with females (HR = 1.85, 95% CI: 1.46–2.34).

## Discussion

This research is unique in its collection of daily symptom presence and severity directly from patients for 28 days during a SARS-CoV-2 infection. Almost 1,000 patients with a self-reported laboratory-confirmed case of COVID-19 were enrolled in January 2021 to complete a daily online survey to assess pre-defined COVID-19 symptom progression over 28 days. The symptoms most frequently reported by patients in this study aligns with existing research [2–4, 8], highlighting fatigue (80%), nasal congestion (73%), headache (72%), cough (66%), aches and pains (57%), and decreased sense of smell (56%) and taste (51%) as the most frequently reported symptoms at enrollment. However, in this study fewer patients experienced fever (34%) compared with previous research [9]. There are a few potential explanations for this difference including enrollment of less severe, non-hospitalized cases. Another consideration is that symptoms must have started within 7 days of enrollment, which could result in patients having a fever prior to enrolling in the study. Results from this study may also underestimate total symptom duration as results report duration captured during study enrollment. Symptom duration before enrollment was short (median 5 days), but symptom duration after Day 28 cannot be estimated. Findings from this study of daily symptom sub-study closely mirror findings obtained from the parent CARE registry in terms of population composition (predominantly White females), as well as distribution of symptoms and symptom persistence over time [2].

Of note, only 30% of patients reported complete resolution of symptoms at the end of our 28-day study period. This is lower than often cited by other studies reporting the majority of patients (80–87%) experience symptom resolution within a month [6, 10]. Fatigue, nasal congestion, cough, and decreased sense of smell and taste were the most persistent symptoms. Patients who do not have complete resolution of symptoms after 30 day study period are known to progress to post-acute sequelae of SARS-CoV-2 infection (PASC), also known as long COVID with studies showing varying burden not limited to respiratory conditions, nervous system, mental health, and fatigue [11–13].

This study captured the patient experience with symptomatic COVID-19 outside of a hospital setting, providing rapid insights that informed development of secondary endpoints and sample size requirements for a clinical trial investigating oral COVID-19 treatment in development during a time when accessibility of information and participation of trials were difficult due to quarantine mandates. Results from this study also highlight the value of this methodology into rapidly generating insights to characterize clinical symptoms to inform both the medical community and clinical trial design and endpoints. Direct-to-patient online study design allowed capturing of daily patient experience that is not reflected in most other data sources, such as medical electronic medical records or administrative claims data. The online survey design, which minimized patient burden, along with payments for time and effort to participate in the study resulted in good study retention, with 75% of patients completing at least 4 of the 7 daily surveys each week. The 4-point scale for COVID-19 symptom severity has been supported by focus groups. Findings are in line with those of clinical trials and other observational studies, which speaks to validity of self-reported data [2–4, 8].

Our study population captured predominantly white, females, with an average age of 40 years, a demographic combination known to frequently volunteer in online surveys [13]. Findings thus may not be generalizable to the broader population. Recruitment was done through social media and this study may not have been accessible to those without internet or unable to enter information electronically. In addition, participants with severe symptoms may have been less able to enroll in the study or complete follow-up, resulting in potential underestimation of symptom severity. This study was conducted in January 2021 when vaccines were not widely available and the dominant strain circulating in the US was primarily Alpha.

## Conclusion

The CARE sub-study provided a platform to quickly collect and analyze daily patient-reported COVID-19 symptom data in non-hospitalized, symptomatic adults with confirmed COVID-19 infection. The study highlights the value of patient-reported data to provide clinicians and patients with important and rapid insights to COVID-19 disease and symptom progression and the potential of using real-world data to inform clinical trial design and endpoints.

### Electronic supplementary material

Below is the link to the electronic supplementary material.

Supplementary Material 1

## Data Availability

The datasets generated and/or analyzed during the current study are not publicly available to maintain patient privacy, but deidentified data may be made available from the corresponding author after review of request.

## List of abbreviations

CARE: COVID-19 Active Research Experience
CI: Confidence Interval
COVID-19: Coronavirus disease 2019
BMI: body mass index
HR: hazard ratio
IQR: interquartile range
